# Glucocorticoids may compromise the effect of gefitinib in non-small cell lung cancer

**DOI:** 10.18632/oncotarget.13185

**Published:** 2016-11-07

**Authors:** Hsian-Yu Wang, Yu-Ling Chang, Chun-Chun Cheng, Min-Wu Chao, Su-I Lin, Shiow-Lin Pan, Chih-Cheng Hsu, Tsang-Wu Liu, Han-Chin Cheng, Ching-Ping Tseng, Shih-Jen Liu, Hui-Ju Tsai, Hsing-Yi Chang, John T.-A. Hsu

**Affiliations:** ^1^ Institute of Biotechnology and Pharmaceutical Research, National Health Research Institutes, Miaoli County, Taiwan; ^2^ Institute of Molecular Medicine and Bioengineering, National Chiao Tung University, Hsinchu City, Taiwan; ^3^ Institute of Population Health Sciences, National Health Research Institutes, Miaoli County, Taiwan; ^4^ The Ph.D. Program for Cancer Biology and Drug Discovery, College of Medical Science and Technology, Taipei Medical University, Taipei City, Taiwan; ^5^ Graduate Institute of Life Sciences, National Defense Medical Center, Taipei City, Taiwan; ^6^ National Institute of Infectious Diseases and Vaccinology, National Health Research Institutes, Miaoli County, Taiwan; ^7^ Institute of Cancer Research, National Health Research Institutes, Miaoli County, Taiwan; ^8^ Institute of Bioinformatics and Systems Biology, National Chiao Tung University, Hsinchu City, Taiwan; ^9^ Miaoli General Hospital, Ministry of Health and Welfare, Miaoli County, Taiwan; ^10^ Graduate Institute of Immunology, China Medical University, Taichung City, Taiwan; ^11^ Division of Biostatistics and Bioinformatics, Institute of Population Health Sciences, National Health Research Institutes, Miaoli County, Taiwan; ^12^ Department of Public Health, China Medical University, Taichung, Taiwan, Taichung City, Taiwan; ^13^ Department of Pediatrics, Feinberg School of Medicine, Northwestern University, Chicago, IL, USA; ^14^ Institute of Public Health, National Yang-Ming University, Taipei City, Taiwan

**Keywords:** NSCLC, EGFR, TKI, glucocorticoids, national health insurance research database taiwan

## Abstract

The epidermal growth factor receptor (EGFR)-targeting tyrosine kinase inhibitors (TKIs) have shown remarkable benefits in non-small cell lung cancer (NSCLC) patients with drug-sensitive mutations in the *EGFR* gene. Responsive patients are usually continuously prescribed with TKIs until disease progression. Glucocorticoids (GCs) are potent homeostasis maintaining drugs and are frequently used in cancer patients to alleviate discomforts caused by anti-cancer therapies. Several previous studies reported that concomitant use of GCs may compromise the efficacy of chemo-therapeutics in patients with solid tumors. Little is known in the concomitant use of target therapy with GCs in treating NSCLC. In this study, we hypothesized that concomitant use of GCs in EGFR-TKI therapy may be detrimental and addressed this issue using cell cultures and xenograft studies followed by a retrospective population study based on data from the Taiwan national health insurance system. In cell cultures and xenograft studies, GCs were shown to unequally compromise the anti-cancer efficacy of TKIs in both PC9 and NCI-H1975 NSCLC cells models. In the retrospective population study, patients with similar disease status that were co-medicated with GCs had a significantly higher risk of disease progression.

## INTRODUCTION

Lung cancer is the leading cause of cancer deaths in the world [1]. Approximately 85% of lung cancer cases are non-small cell lung cancer (NSCLC), including squamous cell carcinoma, adenocarcinoma, and large cell carcinoma, with the rest being small cell lung cancer [2]. Treatment modalities of NSCLC include surgery, radiation therapy, chemotherapy and target therapy. In the past decade, the epidermal growth factor receptor (EGFR)-targeting tyrosine kinase inhibitors (TKIs) such as gefitinib and erlotinib have shown remarkable benefits in all stages of NSCLC patients with drug-sensitive mutations in the *EGFR* gene [3, 4]. Different from chemotherapeutics, the EGFR-targeting drugs showed much lower side effects with significant anti-cancer efficacy in patients harboring drug-sensitive *EGFR* mutations.

In Taiwan, non-small cell lung cancer (NSCLC) patients have a more than 50% response rate to treatment involving epidermal growth factor receptor (EGFR) tyrosine kinase inhibitors (TKIs) because of a high incidence of somatic activating mutations [3–6]. Consequently, since June 2011, the Taiwan National Health Insurance (NHI) policy has included gefitinib as first-line therapy for NSCLC patients with diagnoses of drug-sensitive mutations in EGFRs. In previous clinical study, combination of gefitinib with chemotherapeutics in advanced NSCLC showed no improved efficacy [7]. When patients are confirmed with drug-sensitive EGFR mutations, gefitinib alone was recommended as the first-line therapy.

However, almost all patients with drug-sensitive *EGFR* mutations who initially responded to gefitinib or erlotinib eventually developed resistance to these target therapeutics and switched to chemotherapy. Thus, researchers have been striving to understand and overcome such a problem [8, 9]. Comparing to new drug development, it is equally important to maximize the efficacy of the existing target drugs.

GCs are useful in management of clinical oncology. They not only benefit to treatment of hematologic malignancy, but also help to reduce the pain and the suffering during cancer therapy [10]. Comedication with GCs as prophylaxes have been highly recommended for alleviation of severe side effects caused by chemotherapeutic drugs [11, 12]. In contrast to chemotherapy, systemic administration of GCs is not a standard practice for managing adverse reactions caused by gefitinib treatment in NSCLCs, with the exception of critical conditions such as interstitial lung disease [13–16]. However, we observed from the NHI claims database that more than a quarter of patients (26.5%) were co-medicated with oral form GCs, including dexamethasone, methylprednisolone and prednisolone, during the course of gefitinib treatment.

The use of GCs as co-medication in NSCLC has long been under debate. Some comprehensive reviews have revealed that concomitant use of GCs and chemotherapeutics would reduce the efficacy of the latter in patients with solid tumors [17–21]. However, the potential effects of GCs in lung cancer patients treated with gefitinib remain unclear. Therefore, the goal of this study was to examine whether concomitant use of GCs might compromise the anti-cancer efficacy of target therapy through cell cultures, xenografts, and a population study. Since long-term randomised trials involving the concomitant use of GCs and gefitinib are not feasible due to potential hazards that may be imposed to patients, we employed a retrospective population study based on data from the Taiwan national health insurance system to assess the effects of GCs in gefitinib treated NSCLC patients.

The National Health Insurance Research Database (NHIRD) in Taiwan encompasses the entire 23 million residents; being one of the largest nationwide population database in the world. Since 1995, the NHI program has provided universal coverage of more than 99% of the population in Taiwan. Owing to its enormous sample size, the NHIRD permits a wide range of study design, such as drug adverse effects and risks of disease, drug safety and efficacy, drug prescription and utilization patterns, prognosis and health outcome researches [22].

In this study, the potential hazards of concomitant administration of GCs with gefitinib for NSCLC treatments were assessed in cell culture, xenograft models, and patients with drug-sensitive EGFR mutations that were covered by NHIRD to receive gefitinib as the first-line therapy.

## RESULTS

### Effectiveness of EGFR-TKIs was reduced by treatment with GCs in drug sensitive NSCLC cells

**Figure 1:**
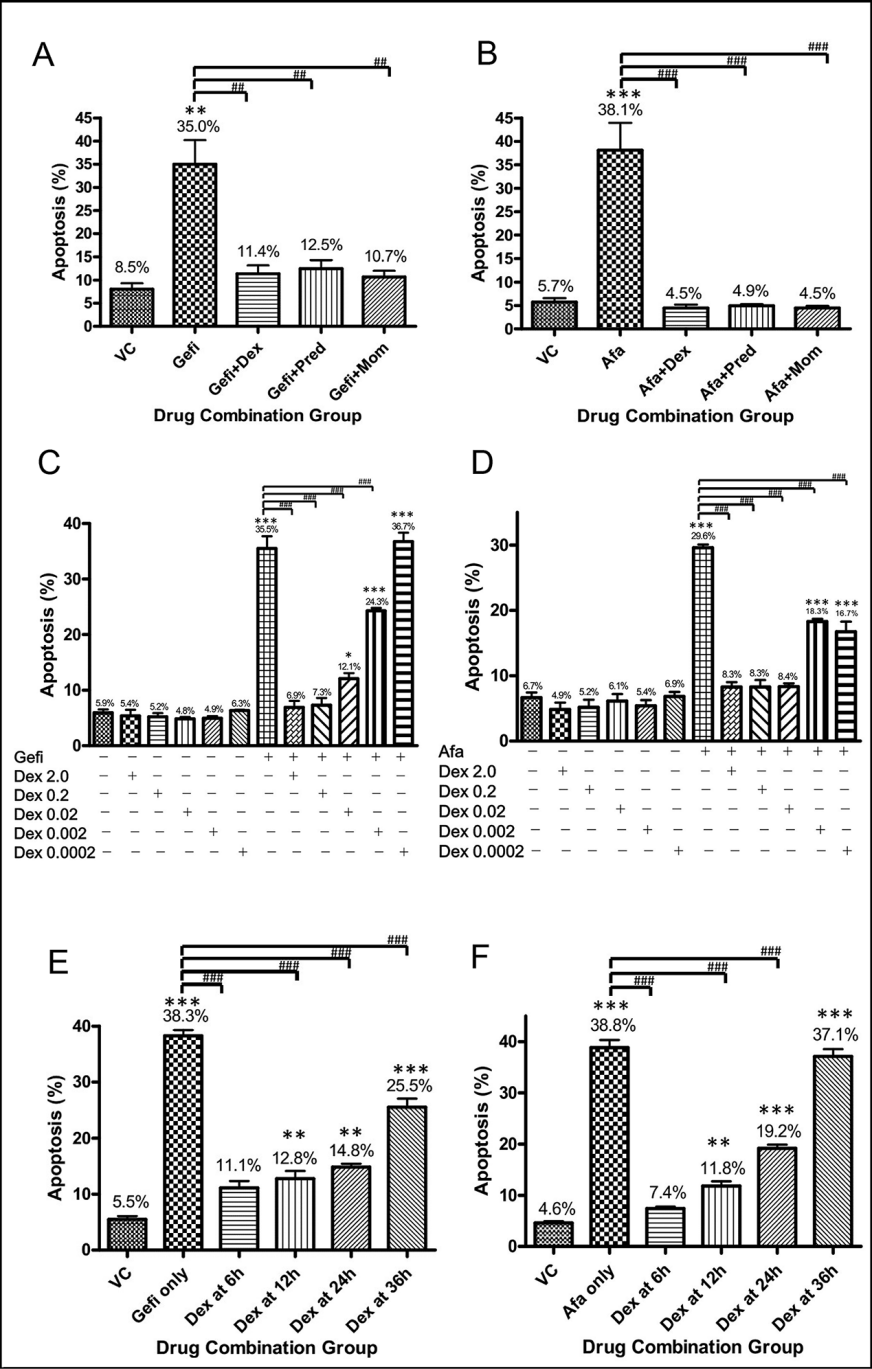
Cell apoptosis results of NSCLC cells treated with an EGFR-TKI alone or in combination with a GC (**A**) PC9 (EGFR^exon19del E746-A750^) cells were treated with vehicle control (DMSO), 200 nM gefitinib alone, or gefitinib and a GC (200 nM gefitinib + 1 μM dexamethasone; 200 nM gefitinib + 1 μM prednisolone; 200 nM gefitinib + 1 μM mometasone). Cell apoptosis was evaluated by apoptosis assay after 48 hours of drug treatment. The percentages of apoptotic cells in each treatment group are shown. Three repeating assays were performed. **p < 0.01 compared to VC; ^##^*p* < 0.01. (**B**) NCI-H1975 (EGFR^L858R/T790M^) cells were treated with the drug combinations described for panel (A) where 500 nM afatinib was used instead of gefitinib. Three repeating assays were performed. ***p < 0.001 compared to VC; ^###^*p* < 0.001. (**C**) PC9 cells were treated with vehicle control (DMSO), 200 nM gefitinib alone, or gefitinib and Dex in five concentrations (2.0 μM, 0.2 μM, 0.02 μM, 0.002 μM and 0.0002 μM). Apoptosis assay was performed after 48 hours of first drug treatment. The percentages of apoptotic cells in each treatment group are shown. Three repeating assays were performed. *p < 0.05 and ***p < 0.001 compared to VC; ^###^*p* < 0.001. (**D**) NCI-H1975 cells were treated with the drug combinations as described in panel (C) where 500 nM afatinib was used instead of gefitinib. Three repeating assays were performed. ***p < 0.001 compared to VC; ^###^*p* < 0.001. (**E**) PC9 cells were treated with vehicle control (DMSO) or gefitinib at 200 nM (IC_80_). After the indicated times—6, 12, 24, and 36 hours—Dex (1 μM in final concentration) was added to the cell culture media. Apoptosis assay was performed after 48 hours of first drug treatment. The percentages of apoptotic cells in each treatment group are shown. Three repeating assays were performed. **p < 0.01 and ***p < 0.001 compared to VC; ^###^*p* < 0.001. (**F**) NCI-H1975 cells were treated with the drug combinations as described in panel (E) where 500 nM afatinib was used instead of gefitinib. Three repeating assays were performed. **p < 0.01 and ***p < 0.001 compared to VC; ^###^*p* < 0.001. Abbreviations: VC, vehicle control; Gefi, gefitinib only; Dex, dexamethasone; Pred, prednisolone; Mom, mometasone, Afa: afatinib.

To determine whether GCs reduced the sensitivity of NSCLC cells to EGFR-TKIs, we treated PC9 cells (EGFR^exon19del E746-A750^) and NCI-H1975 cells (EGFR^L858R/T790M^) with gefitinib and afatinib, respectively, either alone or in combination with a GC. PC9 cells are very sensitive to gefitinib (IC_50_ = 60 nM, IC_80_ = 200 nM), whereas NCI-H1975 cells are sensitive to afatinib but not gefitinib, owing to a second mutation on EGFR i.e., a substitution of methionine for threonine at position 790 (T790M) (IC_50_ = 300 nM, IC_80_ = 500 nM) [23]. The cells were treated with the respective EGFR-TKIs at their IC_80_ values: 200 nM for gefitinib treatment of PC9 cells and 500 nM for afatinib treatment of NCI-H1975 cells. Cells were treated with TKIs alone or in combinations with either, dexamethasone (Dex), prednisolone or mometasone. An Annexin V-propidium iodide staining followed by flow cytometry analysis was employed to assay apoptosis of the cells (Figure 1). As can be seen in Figure 1A and 1B, approximate 35% of the PC9 cells were in the apoptotic state after treatment with gefitinib alone, whereas only 8.5% of the cells were apoptotic after treatment with vehicle control. Strikingly, the percentage of apoptotic PC9 cells dropped substantially when any of the GCs was combined with gefitinib, and the otherwise sensitive NCI-H1975 cells became insensitive to afatinib when it was combined with a GC. Another cell line that is sensitive to gefitinib, NCI-H3255 (EGFR^L858R^), was also used to determine the anti-apoptotic effects of Dex. In NCI-H3255 cells, the percentage of apoptotic cells upon gefitinib treatment was reduced by Dex (Supplementary Figure S1).

To further investigate the dose effects of GC, the very commonly used Dex was serially diluted and combined with TKIs in cell treatment. Figure 1C and 1D showed that a very low amounts of Dex (0.02 μM) was sufficient to dramatically inhibit TKIs induced apoptosis in cell culture test. The anti-apoptotic effect of Dex remained the same even when Dex was added to cells 24 hours after the addition of TKI, indicating that a brief exposure to Dex may still reduce the efficacy of EGFR-TKIs in the treatment of NSCLC cells (Figure 1E, 1F).

### The anti-apoptosis effectiveness of GCs were blocked by glucocorticoid receptor inhibitor, RU486, in EGFR-TKIs treated NSCLC cells

**Figure 2:**
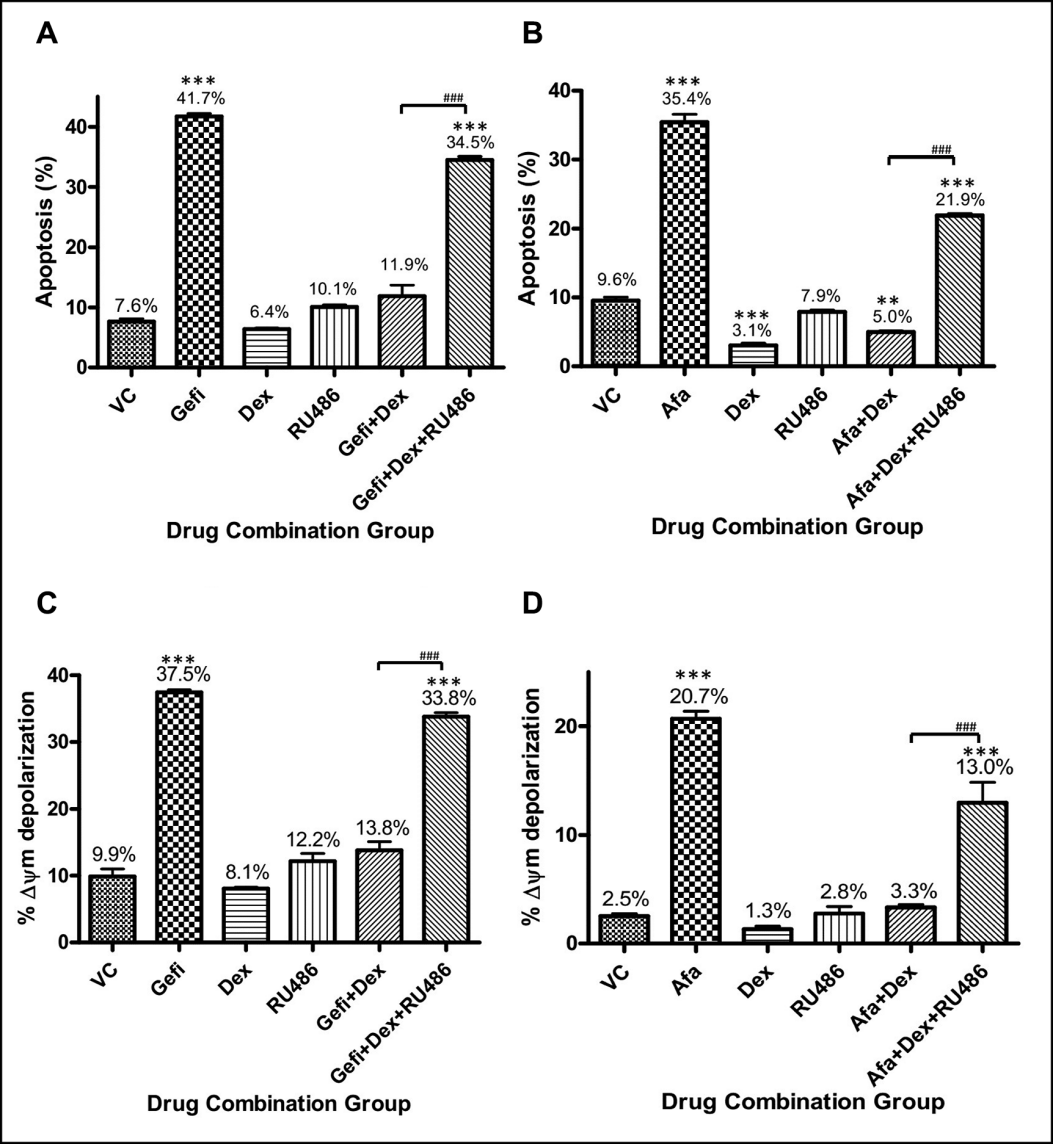
Antagonism of Dex on gefitinib-induced apoptosis in NSCLC cells (**A**) PC9 (EGFR^exon19del E746-A750^) cells were treated with vehicle control (DMSO), gefitinib at 200 nM (IC80), or gefitinib with Dex (1 μM) or RU486 (1 μM). Cell apoptosis was evaluated by apoptosis assay after 48 hours of drug treatment. The percentages of apoptotic cells in each treatment group are shown in the histogram. Three repeating assays were performed. ***p < 0.001 compared to VC; ^###^*p* < 0.001. (**B**) NCI-H1975 (EGFR^L858R/T790M^) cells were treated with the drug combinations described for panel (A), except the 200 nM gefitinib was replaced with 500 nM afatinib. Three repeating assays were performed. **p < 0.01 and ***p < 0.001 compared to VC; ^###^*p* < 0.001. (**C**) PC9 cells were treated with drugs as described in panel (A) for 48 hours and then analyzed for mitochondria membrane potential (MMP), as indicated by % Δψm. Three repeating assays were performed. ***p < 0.001 compared to VC; ^###^*p* < 0.001. (**D**) NCI-H1975 cells were treated with drugs as described in panel (B) for 48 hours and then analyzed for mitochondria membrane potential (MMP), as indicated by %Δψm. Three repeating assays were performed. ***p < 0.001 compared to VC; ^###^*p* < 0.001. Abbreviations: VC, vehicle control; Gefi, gefitinib only; Dex, dexamethasone.

In a previous study, glucocorticoid receptor (GR) was suggested as an important mediator for disruption of death signaling pathways by cisplatin [24]. To explore the underlying mechanism, a potency GR inhibitor, RU486 (mifepristone), was employed to examine whether Dex exerted its anti-apoptotic effects in gefitinib-treated PC9 cells through the action of GR. When RU486 combined into the groups of Dex and TKIs, the Dex induced anti-apoptosis effect was compromised in both NSCLC cell-lines (Figure 2A, 2B). The antagonistic effect of Dex was likely to be exerted through the action of GR.

Since the loss of mitochondria membrane potential (MMP) is a hallmark of intrinsic apoptosis, we examined whether the anti-apoptotic effects of Dex were mediated through the regulation of MMP (Δ*ψ*_m_). PC9 and NCI-H1975 cells were treated with each drugs and their combinations in a manner similar to that as in Figure 2A and 2B. Evidently, the MMP was severely compromised in PC9 cells treated with gefitinib and in H1975 cells treated with afatinib, as compared to cells treated with vehicle control (Figure 2C, 2D). The addition of Dex could fully antagonize the effect of the TKIs on MMP depolarization.

### Dex compromised the anti-cancer efficacy of gefitinib in NSCLC xenografted animal model

**Figure 3:**
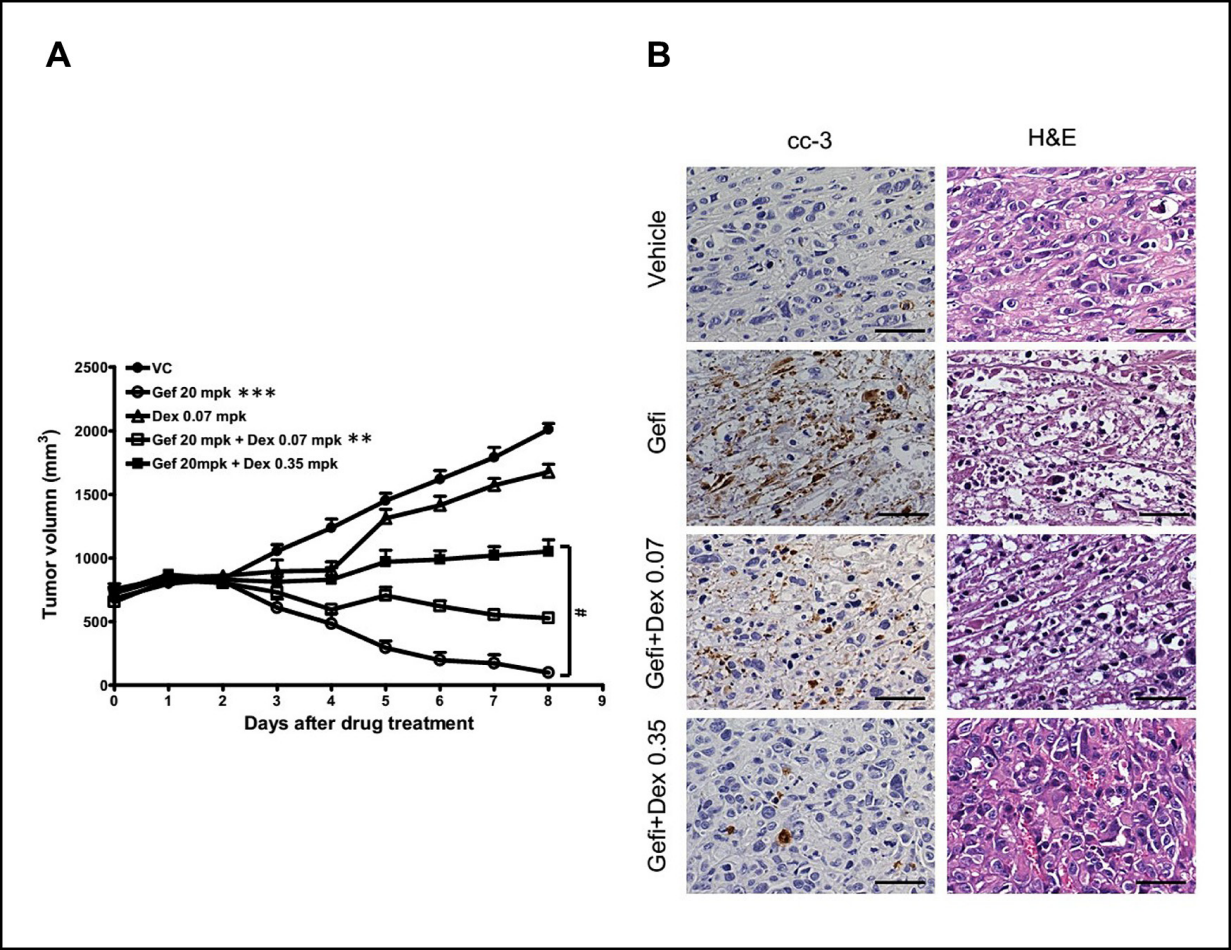
Combined treatment of gefitinib and Dex inhibited cell death in the PC9-xenograft mouse model (**A**) SCID mice were inoculated with PC9 (EGFR^exon19del E746-A750^) cells (5 × 10^6^) subcutaneously at the abdominal site. When tumor volumes reached 800 mm^3^, the vehicle control, gefitinib (Gefi: p.o. 20 mg/kg) alone or in combinations with Dex (p.o. 0.07 mg/kg or 0.35 mg/kg) were orally administered every day for a total of 10 days. Data are means ± SEM. (*n* = 4). **p < 0.01 and ***p < 0.001 compared to VC; ^#^*p* < 0.05. (**B**) After drug exposure for 3 days, tumor cell apoptosis was characterized by cytoplasmic shrinkage and nuclear chromatin condensation in H&E staining and were positive for cleaved caspase 3 positive cells (shown as brown). Scale bars, 50 μm. cc-3, cleaved caspase 3.

To determine whether Dex interfered the anti-tumor effects of gefitinib in mice xenograft, Dex (0.07 or 0.35 mg/kg) was orally co-administered with gefitinib (20 mg/kg) into tumor-bearing mice every day when PC9-xenographed tumor size reached 800 mm^3^ in mice. As shown in Figure 3A, administration of gefitinib alone could effectively shrink the tumor size; all tumors disappeared after 10 days of gefitinib treatment. In contrast, the anti-tumor effects of gefitinib were reduced when co-administered with Dex in a dose-dependent manner. In the 0.07 mg/kg arm, the sizes of tumors decreased in a much slower rate than those in mice receiving gefitinib alone. In the 0.35 mg/kg arm, the anti-cancer efficacy of gefitinib was further reduced. Since early apoptotic response is an important indicator for the anti-cancer efficacy, tumors were taken out of the animals for analysis after 3 days of daily drug treatment. In Figure 3B, it is clear that the gefitinib-triggered caspase 3 mediated apoptosis could be dramatically inhibited by Dex.

The anti-apoptotic effects of Dex were also observed in gefitinib-resistant NCI-H1975 cells (EGFR^L858R/T790M^) grown in nude mice. In Supplementary Figure S2, the growth of NCI-H1975 xenograft tumors (300 mm^3^) could be inhibited with afatinib (20 mg/kg). When Dex were co-administered with afatinib (*p* < 0.05), the efficacy of afatinib was significantly compromised without difference in body weight changes (Supplementary Figure S2A, S2B). It is evident that the afatinib-induced apoptosis in tumors was also inhibited as characterized by less cytoplasmic shrinkage and nuclear chromatin in H&E staining and less cleaved caspase 3 positive cells in mice receiving afatinib and Dex concomitantly (Supplementary Figure S2C).

Interestingly, in either PC9 or NCI-H1975 xenograft model, the Dex only treatment groups showed mild tumor inhibition results. This was compatible with previous studies, GC monotherapy limited tumor growth in some lung cancer cells [21].

### Population data

**Figure 4:**
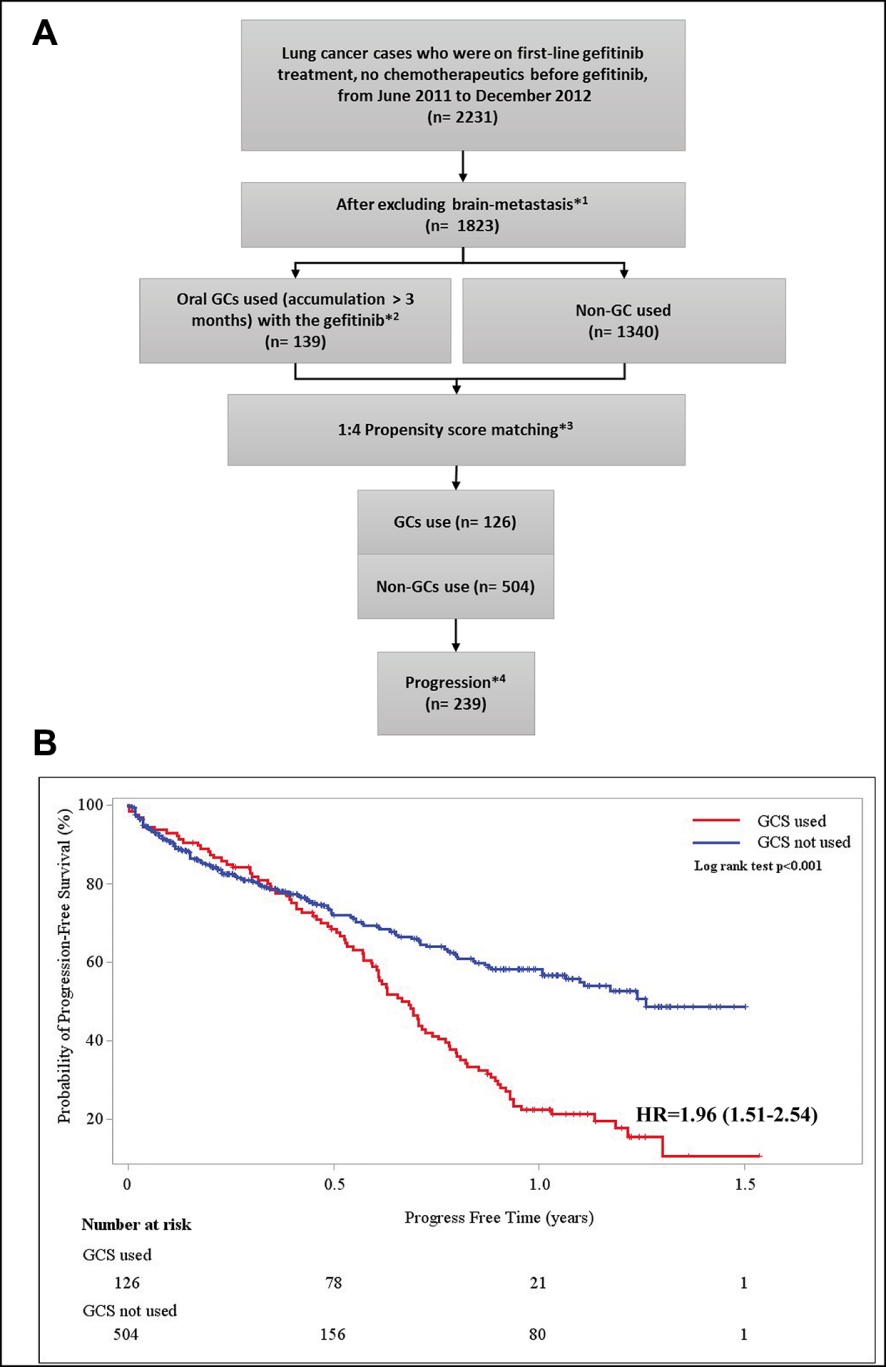
Population data analysis scheme and progression free survivals (years) for patients using gefitinib only or in combination with GCs (**A**) Flow scheme for analyzing the effects of GCs comedication using the Taiwan NHI claim database. *^1^Brain-metastasis (ICD-9-CM 198.3) was excluded because use of GCs was recommended management for such disease status. *^2^A group of cases (*n* = 139) who received oral GCs (dexamethasone, methylprednisolone or prednisolone) for more than 3 months during gefitinib therapy was identified. *^3^Propensity score was calculated using gender and age, as well as disease status including diabetes, hypertensive heart disease, kidney disease, respiratory conditions (including chronic obstructive pulmonary disease, asthma, and bronchitis), metastasis and endotracheal intubation. *^4^ Due to the nature of the NHI claim database, in this observational study, patients with progression was defined as: prescription of a new chemotherapeutic drugs or death. (**B**) Kaplan-Meier analysis of probability of progression-free in gefitinib alone or concomitant use with oral GCs during gefitinib therapy. HR shows the hazard ratio and the 95% confidence intervals (CIs) of the GCs comedication group compared to the non-GCs used group.

In the retrospective study, information from the NHI claims database was employed. Among the NSCLC patients from June 2011 to December 2012, we found that 2,231 patients with drug-sensitive EGFR mutations have been treated with gefitinib as first-line therapy, without any chemotherapeutics before. According to the latest Taiwan Cancer Registry report, these study cases were thus largely composed of stage IV NSCLC patients [25]. We excluded all the patients with brain metastasis which usually received GCs to relieve pain or edema. The injection form of GCs was also excluded due to its possibility of emergency use. Only the patients prescribed with oral forms of GCs were isolated for the following analysis. To further wash out the potential bias caused by the emergency use in the gefitinib treated patients, we extracted the study objects who received accumulated GCs prescription for more than 3 months. To further reduce possible confounding effects caused by covariates such as metastasis, endotracheal intubation, respiratory diseases (including chronic obstructive pulmonary disease, asthma, and bronchitis) and diabetes, propensity score matching was applied. Of note, the flow scheme related to the selection of study subjects for this study is shown in Figure 4A.

After matching, no significant difference was found in disease conditions between cohorts with or without comedication with GCs (Table 1). Although it is not possible to control un-observed confounding factors, that no difference between the cohorts in initial stage (1–4 months) in the Kaplan-Maier analyses indicates similar disease conditions between the two cohorts on the index date (Figure 4B). After the initial stage, the probability of progression-free (PF) became significantly higher in the gefitinib-only group than those in the gefitinib+GC groups. Cox proportional hazard model was employed to assess excess risk of the subjects using GC comedications. The hazard ratio (HR) and the 95% confidence intervals (CIs) of the GCs comedication group was 1.96 (1.51–2.54) (Figure 4B).

**Table 1:** Matched variables in GC drug-use and non-use patient

	Before matching			After matching		
	Not used	Used	*p*	Not used	Used	*p*
*N*	1340	139		504	126	
Progressed	77 (28.1)	104 (74.8)	< 0.001	143 (28.4)	96 (76.2)	< 0.001
Age (mean ± SD), years	68.34 ± 12.48	64.60 ± 12.88	< 0.001	64.82 ± 13.01	64.42 ± 12.39	0.75
Male gender (N, %)	528 (39.4)	59 (42.5)	0.49	214 (42.5)	51 (40.5)	0.69
Female gender (N, %)	812 (60.6)	80 (57.5.)		290 (57.5)	75 (59.5)	
Diabetes (N, %)	213 (15.9)	22 (15.8)	0.98	63 (12.5)	17 (13.5)	0.76
Hypertensive heart disease (N, %)	65 (4.9)	4 (2.9)	0.29	17 (3.4)	3 (2.4)	0.57
Kidney disease (N, %)	58 (4.3)	6 (4.3)	0.99	16 (3.2)	5 (4.0)	0.66
Chronic respiratory conditions (N, %)	504 (37.6)	65 (46.8)	0.03	230 (45.6)	61 (48.4)	0.58
Metastasis (N, %)	955 (71.3)	128 (92.1)	< 0.001	461 (91.5)	115 (91.3)	0.94
Endotracheal Intubation (N, %)	31 (2.3)	19 (13.7)	< 0.001	16 (3.2)	6 (4.8)	0.39

Data are number of patients (%), unless otherwise stated. Diabetes, ICD-9-CM 250; hypertensive heart disease, ICD-9-CM 402–404; kidney disease, ICD-9-CM 580–588; chronic respiratory conditions (including chronic obstructive pulmonary disease, asthma, and bronchitis), ICD-9-CM 490–519; metastasis, ICD-9-CM-196-198. endotracheal intubation, NHI operation code 47031C.

## DISCUSSION

It has been known that concomitant use of glucocorticoids (GCs) and chemotherapeutics may reduce the efficacy of the latter in patients with solid tumors. Keith *et al*. reviewed 54 randomized controlled trials in which GCs were used in cancer patients and found that lung cancer patients receiving both chemotherapeutics and GCs had worse outcomes than those who received chemotherapeutics alone [17]. This current translational study integrated *in vitro* cell culture, xenograft animal models, and a population study to assess the harmful effects of concomitant use of GCs and gefitinib on NSCLC treatments. All results pointed to the same direction. That is, efficacy of gefitinib could be greatly compromised by the often co-administered GCs, and this GCs induced anti-apoptosis of EGFR-TKI-treated NSCLC cells possibly mediated by GR. It is interesting to note that Dex alone had slight inhibition of tumor growth in tumor xenografts (Figure 3 and Supplementary Figure S2). It is likely that such minor effects is due to inhibition of cell growth through cell cycle regulators, e.g., CDK2, CDK4, and cyclin D1 etc., as suggested by Greenberg and colleagues [26].

Results from previous clinical studies indicated that EGFR TKIs such as gefitinib and afatinib should be considered as first-line treatment options for patients with mutation-positive *EGFR* [27, 28]. In 2013, afatinib was approved as a first-line treatment for patients with metastatic lung adenocarcinoma because of the finding that afatinib led to greater PFS when compared to the standard chemotherapy, cisplatin and pemetrexed [29]. Most patients who initially respond to the TKI therapy inevitably relapse with the median PFS ranging from 8.4 months (WJTOG3405, randomized phase III trial) to 13.7 months (LUX-Lung 6, randomized phase III trial). It has been intensively investigated whether novel agents or treatment modalities can prolong the PFS for this subset of NSCLC patients receiving EGFR TKIs. Results from this study strongly suggest a new direction; that is, cautious uses of GCs may avoid undesirable shortening of PFS in NSCLC patients receiving TKI treatments.

The population data in this study was derived from the NHIRD encompassing the entire 23 million national population [22]. Retrospective studies employing information from NHIRD might be the best way to reveal potential detrimental effects of this kind since it is unlikely to justify prospective clinical trials for assessing potential hazardous effects of GCs. By realizing the possible confounding factors, in this study, we focused on oral form GCs which were administrated for more than 3 months and eliminated the brain-metastasis patients that recommended GCs as therapeutics. Furthermore, we strived to minimize possible confounders by propensity score matching between patients. After the differences between the groups were minimized, the risk of progression remained significantly higher in the gefitinib+GC groups than the gefitinib only group. The most common side effects of EGFR TKIs such as gefitinib include epidermal reactions, diarrhea, nausea and vomiting [30]. Personalized medications may be needed for alleviation of the adverse reactions in different patients. For the common skin rashes, topical GCs were recommended for mild (NCI-CTC grade-1) and moderate (NCI-CTC grade-2) reactions. Systematic administration of GCs would be only needed for severe reactions (NCI-CTC grade-3) [31].

The current retrospective study using the NHIRD did suffer from several limitations. First, the potency of GCs used was not available in the NHIRD. On the NHIRD drug list, there are more than 400 kinds of GCs drugs, with varied potency and dosage, and therefore converting the dosages and duration into the same units would be difficult. Second, cancer staging information, prognostic variables such as disease stage, performance status, weight loss and histology were not available in the NHIRD. However, the latest Taiwan Cancer Registry report showed that there were 1,409 cases of lung cancer applied for target therapy in 2012, and 1,287 (91.5%) were in stage IV [25]. As such, our study subjects would very likely compose of cases of mostly stage IV NSCLC. Third, the relevant data for NSCLC patients receiving erlotinib or afatinib in the NHIRD have not been released for research purpose at the time of this study although prescription of erlotinib or afatinib was also approved by NHI for qualified NSCLC patients. Despite these limitations, the population data provides added value to support the conclusion of cell-base and xenograft model results.

Newer generation of EGFR TKIs such as afatinib, rociletinib (CO-1686), and AZD9291 have shown great promises [32, 33]. For AZD9291 and CO1686, the induction of apoptosis in NCI-H1975 cells upon treatment with each different TKI was also greatly suppressed by Dex (Supplementary Table S1). Taken together, concomitant use of a GC in alleviating side effects in NSCLC patients treated by TKIs should be greatly cautioned and future investigations on signal transduction studies will certainly be needed for better understanding the impact of GCs on inhibition of EGFR TKIs.

## MATERIALS AND METHODS

### Ethics statement

All the works performed in animals were approved by the Institutional Animal Care and Use Committee (IACUC) respectively, and were carried out in accordance with the approved guidelines. For PC9 xenograft mice model, the protocol was approved by the IACUC at the National Health Research Institutes (approval no. NHRI-IACUC-103020A), and the protocol of the NCI-H1975 xenograft mice model was approved by the IACUC at the Taipei Medical University (approval no. LAC-2013-0139). For population data study, the protocol design of the data from National Health Insurance Research Database (NHIRD) was approved by the ethical committee of the National Health Research Institutes (approval code is EC 1020904-E). This is an insurance claim data and all the identifiers were scrambled. No individual can be identified. Both cell lines used in the current study can be obtained commercially and they were classified as the lowest risk by the institutional review board of National Health Research Institute. The ethics approval was not required for the use of these cell lines.

### Cell culture and reagents

We used two types of NSCLC cells with respective *EGFR* mutations for this study: PC9 cells (EGFR^exon19del E746-A750^) and NCI-H1975 cells (EGFR^L858R/T790M^). The PC9 cells were kindly provided by Dr. Pan-Chyr Yang at National Taiwan University, and the NCI-H1975 cells were obtained from the American Type Culture Collection (Manassas, VA). PC9 cells are very sensitive to gefitinib (PC9: IC_50_ = 60 nM, IC_80_ = 200 nM), whereas NCI-H1975 cells are sensitive to afatinib (IC_50_ = 300 nM, IC_80_ = 500 nM) but not gefitinib, owing to a second mutation on *EGFR*, i.e., a substitution of methionine for threonine at position 790 (T790M) [23]. Cells were maintained as previously described [34]. All cells were maintained in RPMI 1640 growth medium (Invitrogen, Carlsbad, CA) containing 10% fetal bovine serum (Gibco, Invitrogen), penicillin, and streptomycin (Invitrogen) in humidified 5% CO2 at 37°C. The treatment drugs gefitinib (Ryss Lab, Inc., Union City, CA), afatinib (LC Laboratories, Woburn, MA), dexamethasone, prednisolone, mometasone and mifepristone (Sigma-Aldrich, St. Louis, MO) were commercially obtained. Stock solutions (10 mM) of all chemicals were prepared in DMSO.

### Apoptosis assay

PC9 or NCI-H1975 cells (2 × 10^5^ cells/per well) were seeded in 6-well plates and cultured overnight. Drugs were diluted in culture medium at the desired concentrations and were applied to cells. After drug treatment for 48 hours, all cells were trypsinized, washed with PBS, and resuspended in binding buffer (0.01 M HEPES, pH 7.4; 0.14 M NaCl; 2.5 mM CaCl_2_) on ice. Annexin V-FITC (BD Pharmingen, San Diego, CA) and propidium iodide (Sigma-Aldrich, St. Louis, MO) were then used to stain the apoptotic cells. Cell samples were analyzed by flow cytometry with a FACS Calibur machine (BD Bioscience, Franklin Lakes, NJ), and apoptosis percentages were analyzed by means of CellQuest Pro software (BD Bioscience).

### Determination of mitochondrial membrane potential

Mitochondrial membrane potential (Δψm) was determined by BD^TM^ MitoScreen Flow Cytometry Mitochondrial Membrane Potential Detection Kit (BD Biosciences, Franklin Lakes, NJ, USA). Briefly, cells were seeded and treated by the indicated drug combination as described above. All the cells were harvested and then followed by centrifugation and stained by JC-1 (5,5′,6,6′-tetrachloro-1,1′,3,3′-tetraethylbenz imidazolcarbocyanine iodide) as per manufacturer's recommendations. The residual JC-1 was removed by centrifugation and the pellet was mixed with 1x assay buffer. The changes of mitochondrial Δψm was analyzed by FACS Calibur flow cytometer according to the dual fluorescence characteristic of the JC-1. When cells are healthy, the mitochondria membrane is polarized, and JC-1 is rapidly taken up by such mitochondria leading to rapidly increased concentration and results in aggregates which show red spectral shift in FL-2 channel. When mitochondria membrane is depolarized, JC-1 leaks out of the mitochondria into the cytoplasm as monomers resulting in a decrease of red fluorescence. The quantified data were expressed as the percent of Δψm depolarization.

### Xenograft murine models

For PC9-xenografted model, 20 female SCID mice (BioLASCO, Taiwan) 6–8 weeks of age were used in this study. Human lung cancer PC9 cells (5 × 10^6^/mouse) were injected into the abdominal region of the SCID mice subcutaneously. Mice were randomly divided into five groups (*n* = 4) when tumor size reach 800 mm^3^, and were administered with gefitinib (20 mg/kg) alone or gefitinib plus dexamethasone (0.07 or 0.35 mg/kg) orally every day for a total of 8 treatments. One animal in each group was sacrificed and tumor was removed for the following analysis. Tumor size was measured by using the formula: tumor size = length × width × width / 2. For NCI-H1975 model, male nude mice (BioLASCO, Taiwan) were 5 week-old and had a body weight ranged from 20 to 24 g at day one of the study. Mice were injected subcutaneously with the same volume of BD Matrigel Matrix HC (BD bioscience, catalog 354248) and NCI-H1975 cells (1 × 10^7^ cell/mouse) into the flank of each animal. When the tumors had grown to the size around 300 mm^3^, mice were divided into four groups (*n* = 10) and received the following treatment daily by oral gavage for 14 days during the study, including vehicle (DMSO), afatinib (20 mg/kg), dexamethasone (0.30 mg/kg) and afatinib (20 mg/kg) co-administered with dexamethasone (0.30 mg/kg) respectively. Tumor size was measured twice weekly and calculated from tumor size = length × width × width / 2. At the end of study, tumors were carefully removed for subsequent analysis.

### Immunohistochemistry in the tumor tissue

Tumors isolated from xenograft mice were embedded in paraffin blocks. Immunostaining was performed on 5-μm thick sections of tumor tissue. Tissue slides were performed microwave antigen retrieval in citrate buffer (pH 6.0) for 10 min prior to peroxidase quenching with 3% H_2_O_2_ in PBS for 10 min. Tissue sections were incubated with anti-cleaved caspase-3 (Cell Signaling, Danvers, MA) at 4°C overnight and then incubated with biotinylated secondary antibodies for 1 h following a washing step with PBS, streptavidin-HRP was applied. Finally, the sections were developed with diaminobenzidine tetrahydrochloride (DAB) substrate for 10 min, and counterstained with hematoxylin.

### Statistical analysis in cell culture and animal studies

Statistical analysis was performed by means of one-way analysis of variance (ANOVA). The results are showed as means ± SEM. A *p-value* less than 0.05 was considered as statistically significant.

### Population data

#### Data source and study cohort

The population data for this study came from the reimbursement medical claims data collected in the NHIRD, derived from the National Health Insurance (NHI) program in Taiwan. Briefly, the NHI program was implemented in 1995, and 98.4% of Taiwan's population of 23 million was enrolled in the program by 2007. The ICD-9-CM (International Classification of Diseases, 9th revision, clinical modification) was used to identify NSCLC patients. Patients who had at least two clinical visits or one hospitalization for lung cancer (ICD-9-CM 162) were considered as confirmed cases.

### Exposure to gefitinib and/or glucocorticoids treatment

Patients who were on first-line gefitinib treatment from June 2011 to December 2012 were selected since the Taiwan NHI policy started to cover the use of gefitinib as first-line therapy for NSCLC in June of 2011. Data after December 2012 has not yet been released by the Ministry of Health and Welfare. According to Taiwan's NHI policy, only patients with drug-sensitive EGFR mutations can be reimbursed for prescription with gefitinib as the first-line therapy. Patients co-medicated with gefitinib with either of the three kinds of GCs, including dexamethasone, methylprednisolone, and prednisolone prescribed in oral form, were extracted from the prescription record. The GCs drug-day was also calculated. Only the patients prescribed with GCs for an accumulation of more than 3 months (84 days) were considered as GCs user.

### Definition of progression

Patients with progression were defined as: (1) switched to prescription of a new chemotherapeutic drug, or (2) death. In practice, if first-line EGFR-TKI-treated NSCLC patients clinically progressed to a worse condition, chemotherapy would be subsequently applied. The following chemotherapeutics (ATC code: L01XA01, L01XA02, L01BC05, L01CD02, L01CB01, L01BA04, L01CD01, L01CA04 and L01BC53) were defined according to the Anatomical Therapeutic Chemical (ATC) classification system [35], and extracted from NHIRD.

### Statistical methods

We computed and compared the distributions of demographic and clinical characteristics of the study subjects between GC-used and non-GC used groups using the chi-square test for categorical variables and F-test for continuous variables, separately. To control for potential confounding effects associated with GCs uses, we had excluded all the patients with brain metastasis (ICD9-CM 198.3), and that the matching for propensity score was applied to minimize the characteristic differences between patients who received or did not receive GCs. A propensity score is the predicted probability of an exposure, say using GCs. When propensity scores are similar, the distribution of observed baseline covariates would be similar between the two groups [36]. A SAS-macro is available for the computation and matching cases and controls [37]. In detail, the propensity score in this study was calculated using gender and age, as well as disease status including metastasis (ICD-9- CM 196-198), endotracheal intubation (NHI operation code 47031C), diabetes (ICD-9-CM 250), hypertensive heart disease (ICD-9-CM 402-404), kidney disease (ICD9-CM 580-588), respiratory diseases, including chronic obstructive pulmonary disease, asthma, and bronchitis (ICD-9-CM 490-519) which recorded before first dose of gefitinib administration. The patients in the gefitinib+GC groups were matched with those in the gefitinib-only group at a ratio of 1:4. After matched by propensity score, there were no significant difference in these disease conditions (Table 1). The Cox proportional hazards model with adjustment of age and sex was used to compare the probability of progression-free in the gefitinib+GC to gefitinib-only groups. The gefitinib-only group served as the reference group. Additionally, we plotted Kaplan-Meier curves for probability of progression-free between the gefitinib-alone group and the concomitant use of studied GCs group over the observation period. *P*-values less than 0.05 were declared to be statistically significant. All analyses were performed using SAS version 8.2 (SAS institute, Cary, NC).

## SUPPLEMENTARY MATERIALS FIGURES AND TABLES



## References

[1] (2015). Organization WH. Cancer.

[2] Breathnach OS, Freidlin B, Conley B, Green MR, Johnson DH, Gandara DR, O’Connell M, Shepherd FA, Johnson BE (2001). Twenty-two years of phase III trials for patients with advanced non-small-cell lung cancer: sobering results. J Clin Oncol.

[3] Paez JG, Janne PA, Lee JC, Tracy S, Greulich H, Gabriel S, Herman P, Kaye FJ, Lindeman N, Boggon TJ, Naoki K, Sasaki H, Fujii Y (2004). EGFR mutations in lung cancer: correlation with clinical response to gefitinib therapy. Science.

[4] Lynch TJ, Bell DW, Sordella R, Gurubhagavatula S, Okimoto RA, Brannigan BW, Harris PL, Haserlat SM, Supko JG, Haluska FG, Louis DN, Christiani DC, Settleman J (2004). Activating mutations in the epidermal growth factor receptor underlying responsiveness of non-small-cell lung cancer to gefitinib. N Engl J Med.

[5] Gazdar AF (2009). Activating and resistance mutations of EGFR in non-small-cell lung cancer: role in clinical response to EGFR tyrosine kinase inhibitors. Oncogene.

[6] Chen YR, Fu YN, Lin CH, Yang ST, Hu SF, Chen YT, Tsai SF, Huang SF (2006). Distinctive activation patterns in constitutively active and gefitinib-sensitive EGFR mutants. Oncogene.

[7] Giaccone G, Herbst RS, Manegold C, Scagliotti G, Rosell R, Miller V, Natale RB, Schiller JH, J Von Pawel, Pluzanska A, Gatzemeier U, Grous J, Ochs JS (2004). Gefitinib in combination with gemcitabine and cisplatin in advanced non-small-cell lung cancer: a phase III trial—INTACT 1. J Clin Oncol.

[8] Jackman D, Pao W, Riely GJ, Engelman JA, Kris MG, Janne PA, Lynch T, Johnson BE, Miller VA (2010). Clinical definition of acquired resistance to epidermal growth factor receptor tyrosine kinase inhibitors in non-small-cell lung cancer. J Clin Oncol.

[9] Kobayashi S, Boggon TJ, Dayaram T, Janne PA, Kocher O, Meyerson M, Johnson BE, Eck MJ, Tenen DG, Halmos B (2005). EGFR mutation and resistance of non-small-cell lung cancer to gefitinib. N Engl J Med.

[10] Walsh D, Avashia J (1992). Glucocorticoids in clinical oncology. Cleve Clin J Med.

[11] Herrstedt J, Roila F, Group EGW (2008). Chemotherapy-induced nausea and vomiting: ESMO clinical recommendations for prophylaxis. Ann Oncol.

[12] Hesketh PJ (2008). Chemotherapy-induced nausea and vomiting. N Engl J Med.

[13] Goto Y, Hojo M, Takeda Y, Kobayashi N, Kudo K (2010). Gefitinib-induced interstitial lung disease-addition of intravenous cyclophosphamide to corticosteroids is a valuable treatment option: A case report. Med Oncol.

[14] Xue X, Xue Q, Liu Y, Pan L, Wang K, Zhang L, Wang N, Yang B, Wang J (2013). Gefitinib in combination with prednisolone to avoid interstitial lung disease during non-small cell lung cancer treatment: A case report. Oncol Lett.

[15] Kataoka K, Taniguchi H, Hasegawa Y, Kondoh Y, Kimura T, Nishiyama O, Imaizumi K, Kawabe T, Kume H, Shimokata K (2006). Interstitial lung disease associated with gefitinib. Respir Med.

[16] Akamatsu H, Inoue A, Mitsudomi T, Kobayashi K, Nakagawa K, Mori K, Nukiwa T, Nakanishi Y, Yamamoto N (2013). Interstitial lung disease associated with gefitinib in Japanese patients with EGFR-mutated non-small-cell lung cancer: combined analysis of two Phase III trials (NEJ 002 and WJTOG 3405). Jpn J Clin Oncol.

[17] Keith BD (2008). Systematic review of the clinical effect of glucocorticoids on nonhematologic malignancy. BMC Cancer.

[18] Herr I, Gassler N, Friess H, Buchler MW (2007). Regulation of differential pro- and anti-apoptotic signaling by glucocorticoids. Apoptosis.

[19] Mattern J, Büchler MW, Herr I (2014). Cell Cycle Arrest by Glucocorticoids May Protect Normal Tissue and Solid Tumors from Cancer Therapy. Cancer Biology & Therapy.

[20] Ge H, Ni S, Wang X, Xu N, Liu Y, Wang X, Wang L, Song D, Song Y, Bai C (2012). Dexamethasone reduces sensitivity to cisplatin by blunting p53-dependent cellular senescence in non-small cell lung cancer. PLoS One.

[21] Taylor KM, Ray DW, Sommer P (2016). Glucocorticoid receptors in lung cancer: new perspectives. J Endocrinol.

[22] Hsing AW, Ioannidis JP (2015). Nationwide Population Science: Lessons From the Taiwan National Health Insurance Research Database. JAMA Intern Med.

[23] Pao W, Miller VA, Politi KA, Riely GJ, Somwar R, Zakowski MF, Kris MG, Varmus H (2005). Acquired resistance of lung adenocarcinomas to gefitinib or erlotinib is associated with a second mutation in the EGFR kinase domain. PLoS Med.

[24] Herr I, Ucur E, Herzer K, Okouoyo S, Ridder R, Krammer PH, DoeberitzM von Knebel, Debatin KM (2003). Glucocorticoid cotreatment induces apoptosis resistance toward cancer therapy in carcinomas. Cancer Res.

[25] Welfare T (2015). Cancer Administration Year Report.

[26] Greenberg AK, Hu J, Basu S, Hay J, Reibman J, Yie TA, Tchou-Wong KM, Rom WN, Lee TC (2002). Glucocorticoids inhibit lung cancer cell growth through both the extracellular signal-related kinase pathway and cell cycle regulators. Am J Respir Cell Mol Biol.

[27] Sebastian M, Schmittel A, Reck M (2014). First-line treatment of EGFR-mutated nonsmall cell lung cancer: critical review on study methodology. Eur Respir Rev.

[28] Health Quality O (2010). Epidermal Growth Factor Receptor Mutation (EGFR) Testing for Prediction of Response to EGFR-Targeting Tyrosine Kinase Inhibitor (TKI) Drugs in Patients with Advanced Non-Small-Cell Lung Cancer: An Evidence-Based Analysis. Ont Health Technol Assess Ser.

[29] Sequist LV, Yang JC, Yamamoto N, O’Byrne K, Hirsh V, Mok T, Geater SL, Orlov S, Tsai CM, Boyer M, Su WC, Bennouna J, Kato T (2013). Phase III study of afatinib or cisplatin plus pemetrexed in patients with metastatic lung adenocarcinoma with EGFR mutations. J Clin Oncol.

[30] Cersosimo RJ (2006). Gefitinib: an adverse effects profile. Expert Opin Drug Saf.

[31] Dhull AK, PG V K (2014). Gefitinib and Dermal Reaction: Uncovering the Path of Management. Austin Journal of Clinical Case Reports.

[32] Sequist LV, Rolfe L, Allen AR (2015). Rociletinib in EGFR-Mutated Non-Small-Cell Lung Cancer. The New England journal of medicine.

[33] Janne PA, Yang JC, Kim DW, Planchard D, Ohe Y, Ramalingam SS, Ahn MJ, Kim SW, Su WC, Horn L, Haggstrom D, Felip E, Kim JH (2015). AZD9291 in EGFR inhibitor-resistant non-small-cell lung cancer. N Engl J Med.

[34] Chiu HC, Chou DL, Huang CT, Lin WH, Lien TW, Yen KJ, Hsu JT (2011). Suppression of Stat3 activity sensitizes gefitinib-resistant non small cell lung cancer cells. Biochem Pharmacol.

[35] WHO (2014). WHO Collaborating Centre for Drug Statistics Methodology, Guidelines for ATC classification and DDD assignment.

[36] Austin PC (2011). An Introduction to Propensity Score Methods for Reducing the Effects of Confounding in Observational Studies. Multivariate Behav Res.

[37] Parsons LS (2006). Ovation research Group, SAS users group (SUGI) paper 165–29. Ovation research Group, SAS users group (SUGI). paper.

