# Supplementary material for: Glucocorticoids may compromise the effect of gefitinib in non-small cell lung cancer
Source: Oncotarget. 2016 Nov 7;7(52):85917–28. doi: 10.18632/oncotarget.13185 (PMC5349885; doi:10.18632/oncotarget.13185)
Supplement: Supplementary file 1 [file oncotarget-07-85917-s001.pdf]

# Glucocorticoids may compromise the effect of gefitinib in non-small cell lung cancer

## Supplementary Materials

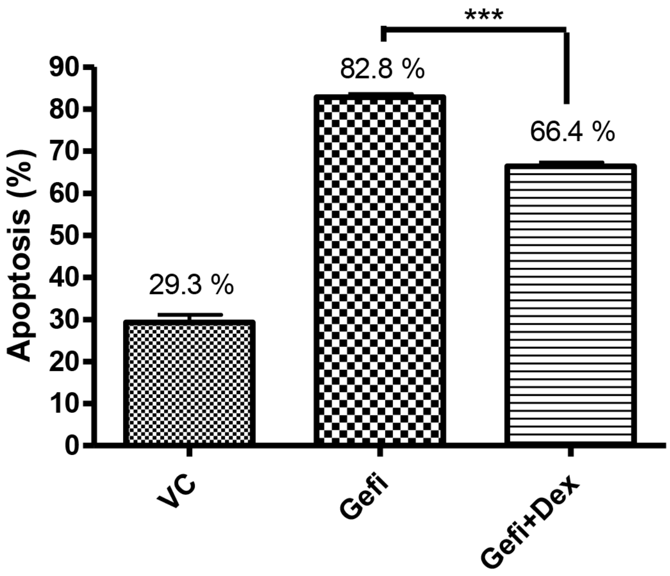


**Supplementary Figure S1: Dexamethasone significantly lowered apoptosis induction in NCI-H3255 cells treated by gefitinib.** NCI-H3255 cells were treated by gefitinib at 90 nM ($IC_{80}$) or in combination with dexamethasone, 1 μM. Three independent repeat tests were performed. Abbreviation: VC, vehicle control; Gefi, gefitinib; Dex, dexamethasone. ***$p < 0.001$.

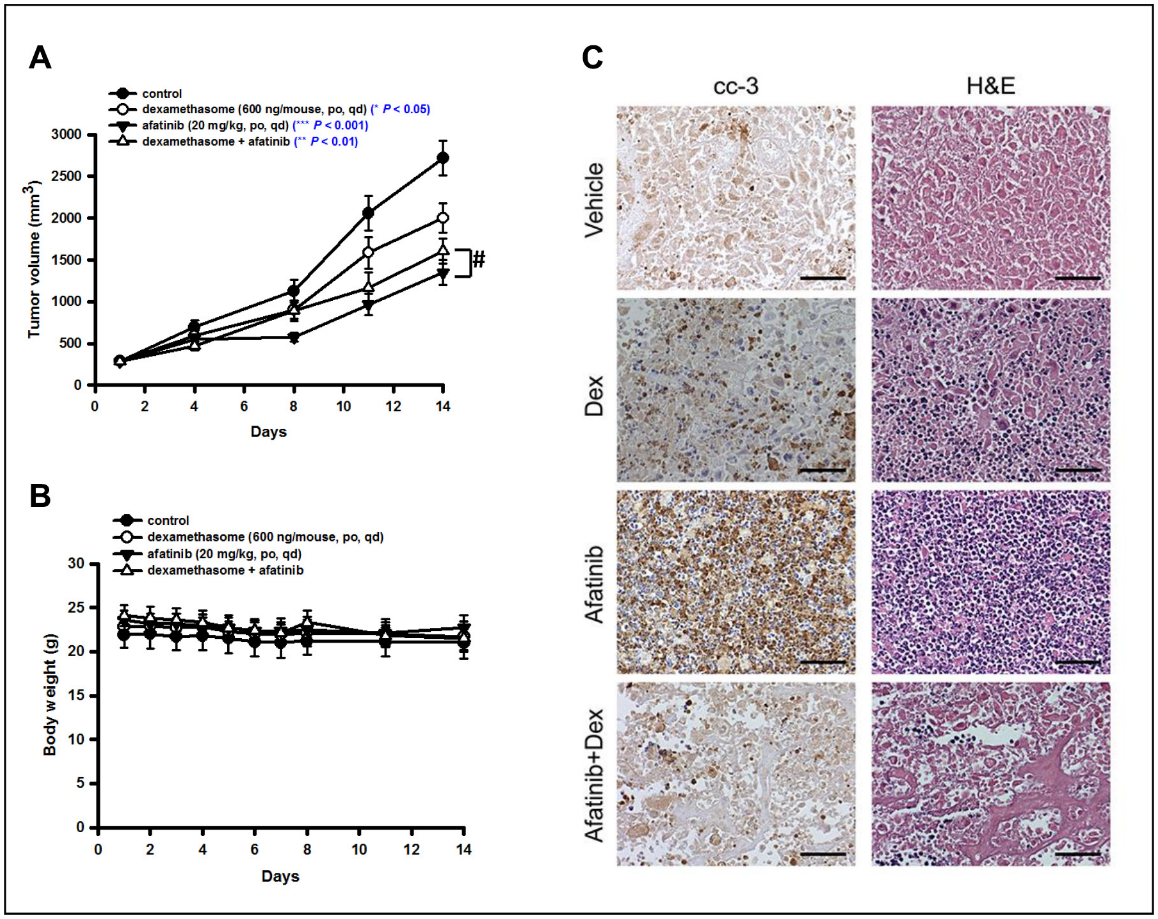


**Supplementary Figure S2: Anti-cancer activity of dexamethasone in combination with afatinib in human non-small cell lung cancer NCI-H1975 xenograft model.** (**A**) Nude mice were daily treated with vehicle (DMSO), afatinib (20 mg/kg), dexamethasone (600 ng/mouse) or afatinib (20 mg/kg) co-administered with dexamethasone (600 ng/mouse) by oral gavage for 14 days. Ten mice per group were used in the xenograft experiment. *$p < 0.05$ and **$p < 0.01$ compared to control; $^{\#}p < 0.05$. (**B**) Body weight of the NCI-H1975tumor bearing mice under the treatment during the study. The data represent mean ± SEM from ten mice in each group. (**C**) Apoptosis in NCI-H1975 xenograft tumors. At the end of 14-day drug treatment, apoptotic cells in tumors were characterized by cytoplasmic shrinkage and nuclear chromatin condensation in H&E staining and were positive for cleaved caspase 3 positive cells (shown as brown). Scale bars, 50 µm. cc-3, cleaved caspase 3.

**Supplementary Table S1: NCI-H1975 cells treated with different EGFR-TKI alone or in combination with dexamethasone (apoptosis %)**

| EGFR-TKI \ Dex. | Non-Dex | Dex (1 μM) |
|---|---|---|
| **Non-TKI** | 9.6 (±1.7)* % | 3.1 (±1.0) % |
| **AZD9291 (1 μM)** | 36.3 (±2.5) % | 5.6 (±1.4) % |
| **CO1686 (1 μM)** | 22.7 (±3.6) % | 5.5 (±0.9) % |

Dex.: dexamethasone.
*mean (±2SD)
